# Improved production of succinic acid from *Basfia succiniciproducens* growing on *A. donax* and process evaluation through material flow analysis

**DOI:** 10.1186/s13068-019-1362-6

**Published:** 2019-02-04

**Authors:** Donatella Cimini, Lucio Zaccariello, Sergio D’Ambrosio, Licia Lama, Giovanna Ruoppolo, Olimpia Pepe, Vincenza Faraco, Chiara Schiraldi

**Affiliations:** 1 Department of Experimental Medicine, University of Campania L. Vanvitelli, Via de Crecchio 7, 80138 Naples, Italy; 2 Department of Environmental, Biological and Pharmaceutical Sciences and Technologies, University of Campania L. Vanvitelli, Via Vivaldi 43, 81100 Caserta, Italy; 3 Institute of Biomolecular Chemistry (ICB), National Research Council, Via Campi Flegrei, 34, 80078 Pozzuoli, Naples Italy; 4 Istituto di Ricerche sulla Combustione (IRC), National Research Council, Piazzale Tecchio 80, 80125 Naples, Italy; 5 Department of Agricultural Sciences, Division of Microbiology, University of Naples Federico II, Via Università 100, 80055 Portici, Naples Italy; 6 Department of Chemical Sciences, University of Naples Federico II, Naples, Italy

**Keywords:** *Basfia succiniciproducens*, *Arundo donax*, Fed-batch, Succinic acid, Pre-pilot scale, Inhibition, Material flow analysis

## Abstract

**Background:**

Due to its wide range of applications in the food, pharmaceutical and chemical fields, microbial synthesis of succinic acid is receiving growing attention, generating already relevant industrial results, as well as fueling constant research for improvements. In order to develop a sustainable process, a special focus is now set on the exploitation and conversion of lignocellulosic biomasses into platform chemicals.

**Results:**

In the present work we used *Basfia succiniciproducens* BPP7 in separated hydrolysis and fermentation experiments with *Arundo donax* as starting material. Fed-batch strategies showed a maximal production of about 37 g/L of succinic acid after 43 h of growth and a productivity of 0.9 g/L h on the pilot scale. Global mass balance calculations demonstrated a hydrolysis and fermentation efficiency of about 75%. Moreover, the application of a material flow analysis showed the obtainment of 88.5 and 52 % of succinic acid, per kg of virgin biomass and on the total generated output, respectively.

**Conclusions:**

The use of fed-batch strategies for the growth of *B. succiniciproducens* on *A. donax* improved the titer and productivity of succinic acid on pre-pilot scale. Process evaluation through material flow analysis showed successful results and predicted a yield of succinic acid of about 30% in a fed-batch process that uses *A. donax* as only carbon source also in the feed. Preliminary considerations on the possibility to achieve an energetic valorization of the residual solid coming from the fermentation process were also carried out.

**Electronic supplementary material:**

The online version of this article (10.1186/s13068-019-1362-6) contains supplementary material, which is available to authorized users.

## Introduction

Due to its recent applications as specialty chemical for the production of biopolymers, and bioplastics in particular, the establishment of sustainable processes for microbial production of succinic acid is receiving great attention [1]. Several biotech companies that appeared in the past few years (e.g. Myriant, Reverdia, BioAmber, and Succinity) established bio-based production platforms that mainly exploit recombinant microorganisms to convert purified sugars into this added value compound [2, 3]. However, as for bio-ethanol production, currently, the identification of strategies that allow conversion of non-food waste materials into platform chemicals is of great value, and bio-based succinic acid production is regarded as one of the fastest growing markets [4]. Diverse lignocellulosic biomasses were used as raw materials for the development of small-scale fermentation processes with natural producers of succinic acid [5–7]; however, extraction of fermentable sugars from complex matrices is often associated with low sugar yields and release of compounds that inhibit microbial growth during hydrolysis [8]. In this work, the dedicated energy crop, *Arundo donax*, was used as source for biological production of succinic acid from *Basfia succiniciproducens* BPP7 [9]. In fact, it was previously shown that this strain is able to grow on (50%) diluted perennial cane in batch processes up to the pilot scale (150 L), also demonstrating the ability to detoxify some of the inhibitory molecules found in the hydrolysate such as hydroxymethylfurfural (HMF), furfural and vanillin [10, 11]. Other examples in literature show the ability of *B. succiniciproducens* to produce succinic acid from various renewable materials. Recently a yield of 0.69 g/g (on consumed sugar) and a productivity of 0.43 g/L h of succinic acid were obtained by growing *B. succiniciproducens* on corn stover in 0.5 L reactors [11]. Besides the potential additional costs for the design of support materials, and the need for process optimization, the production of 45 g/L of succinic acid with a productivity of 0.58 g/L h from nanofiltered spent sulphite liquor was achieved in 0.5 L fed-batch processes with immobilized cells [12].

In the present work fed-batch experiments in anaerobic conditions were conducted on pre-pilot scale bioreactors, growing this promising strain on *A. donax* hydrolysate. A different approach based on a material flow analysis (MFA) method was also established to evaluate the efficiency of the whole process. In particular, the experimental results obtained from the tests of hydrolysis and fermentation, together with those generated by MFA, were combined in order to evaluate the performance of the supposed succinic acid production process (SAPP) also considering the pre-treatment and purification step based on literature data. Moreover, the potential energetic valorization of the residue of the hydrolysis and fermentation processes was also evaluated by ultimate analysis.

## Materials and methods

### Enzymatic hydrolysis of *A. donax*

The lignocellulosic biomass, derived from dedicated crops of *A. donax* and steam pretreated according to Garbero and collaborators [13], was used for the production of fermentable monosaccharides, such as glucose and xylose, for subsequent fermentation experiments. After the treatment, the slurry was diluted in order to have a solid content of about 10% w/v (98 g_drybiomass_/L). The bioconversion process was carried out by slightly changing the previously described protocol [10] with the commercial enzymatic cocktail Novozymes NS22201. Briefly the mixture consisted of 1 L sodium acetate buffer at pH 5.2 per kg of wet lignocellulosic biomass and the final concentration of the applied sodium acetate buffer was 50 mM for an incubation period of 48 h at 45 °C; 140 U of the enzymatic cocktail were added to 1 g of wet biomass and the units, expressed as units of cellulase activity, were determined utilizing the soluble chromogenic substrate carboxymethyl cellulose-Remazolbrillant Blue R (Azo-CM-cellulose, Megazyme, Ireland), calculated from a standard curve constructed with known amounts of cellulase from *Tricoderma* sp. After bioconversion, the liquid residue was removed with a peristaltic pump to separate them from the solids, which were used for the production of succinic acid. Before fermentation experiments the concentration of glucose and xylose released in the process, and present in the liquid fraction, was determined as described in “HPLC quantification of sugars, organic acids and inhibitors” section.

The conversion of cellulose and hemicellulose to glucose and xylose, respectively, as percentage of their theoretical yields from the saccharification of *A. donax* was calculated with the following equations according to [14]:

1 $${\text{Cellulose}}\;{\text{conversion}} \,\left( \% \right) = \frac{{\left[ {\text{Glucose}} \right]}}{{\left[ {\text{Biomass}} \right] \cdot F_{\text{cell}} \cdot 1.11}} \cdot 100$$

2 $${\text{Hemiellulose}}\;{\text{conversion}} \,\left( \% \right) = \frac{{\left[ {\text{Xylose}} \right]}}{{\left[ {\text{Biomass}} \right] \cdot F_{\text{hcell}} \cdot 1.14}} \cdot 100,$$

where [Glucose or Xylose] is the concentrations of glucose and xylose found in the hydrolysates (g/L); [Biomass] is the concentration of dry *A. donax* used during the hydrolytic reaction (g/L); *F*_cell_ and *F*_hcell_ are the fractions of α-cellulose and hemicelluloses in the dry biomass (g/g), respectively [15]; 1.11 and 1.14 are the factors for the conversion of glucans to glucose and xylans to xylose, respectively [16].

### Fermentation experiments

Fermentation experiments were performed on a Biostat D (150 L total volume) with a working volume of 70–80 L (Sartorius Stedim; Melsungen, Germany). The seed culture was inoculated with the *B. succiniciproducens* BPP7 working cell bank and grown for 16 ± 1 h in 0.25 L bottles on MH medium with glucose as C-source and then transferred in a Biostat CT before performing the main cultivation in the Biostat D fermenter. All fermentations were carried out at 37 °C on MH medium containing the following per litre: 5 g yeast extract, 2 g (NH_4_)_2_SO_4_, 0.2 g CaCl_2_∙H_2_O, 0.2 g MgCl_2_∙6H_2_O, 2 g NaCl, 3 g K_2_HPO_4_, 1 mg Na_2_S∙9H_2_O supplemented with 50–60% *A. donax* hydrolysate and run for up to 54 h. The culture was sparged with CO_2_ at 0.1 vvm and agitation speed was set to 100–200 rpm. A constant pH of 6.5 was maintained via automated addition of 25% v/v NH_4_OH and 30% v/v H_2_SO_4_. For the fed-batch phase a concentrated feeding solution containing sugars and yeast extract was added to the broth to prolong growth. The feeding profiles provided addition of about 33–55 g/L of total sugars either pure or diluted in *A. donax* with a rate ranging from 0.2 to 1.2 g/L h. For the duration of all cultivations 50 mL samples were withdrawn from the reactors at regular time intervals for the determination of dry cell weight, substrate consumption and extracellular metabolite production.

The composition of *A. donax* hydrolysate used in the work is the following: glucose 28.9 ± 2.9 g/L, xylose 15.6 ± 1.4 g/L, acetic acid 5.6 ± 0.6 g/L.

The global evaluation of process efficiency considering the conversion of cellulose and hemicellulose to monosaccharides and their fermentation to succinic acid was performed with the following equation:

3 $${\text{Eff}} . \left( \% \right) = \frac{{\left[ {{\text{Succinic}}\;{\text{acid}}} \right] \cdot V_{\text{s}} }}{{\left[ {\left( {\left[ {\text{Cell}} \right] \cdot V_{\text{h}} \cdot 1.11} \right) + \left( {\left[ {\text{Hcell}} \right] \cdot V_{\text{h}} \cdot 1.14} \right) + \left( {\left[ G \right] \cdot V_{{{\text{s}} \cdot }} } \right) + \left( {\left[ X \right] \cdot V_{{{\text{s}} \cdot }} } \right) - \left( {\left[ {G_{\text{a}} } \right] \cdot V_{{{\text{s}} \cdot }} } \right) - \left( {\left[ {\text{Xa}} \right] \cdot V_{{{\text{s}} \cdot }} } \right)} \right] \cdot 1.12}} \cdot 100,$$

where [Succinic acid] is the final concentration of succinic acid in the fermentation broth (g/L); [Cell or Hcell] is the concentration of α-cellulose and hemicellulose in the dry biomass (g/L), respectively, as described by Shatalov and Pereira [15]; *V*_s_ is the volume of supernatant recovered after fermentation; *V*_h_ is the volume of hydrolysate added to the medium; [*G*] and [*X*] are the concentrations of pure glucose and xylose eventually supplemented during fermentation; [*G*_a_] and [*X*_a_] are the concentrations of accumulated glucose and xylose at the end of the FB; 1.12 is the conversion factor of glucose and xylose to succinic acid considering the maximum theoretical yield [17, 18]. 1.11 and 1.14 are the factors for the conversion of glucans to glucose and xylans to xylose, respectively [16]. Conversion of hemicelluloses to glucose was negligible (about 0.6%).

### Shake flasks experiments

Growth inhibition experiments were conducted in 0.25 L bottles filled with 0.25 L of medium at 37 °C and 140 rpm, in a rotary shaker incubator (model Minitron, Infors, Bottmingen, Switzerland). Bottles were sealed with stainless steel headpiece caps and sterile venting filters to insufflate CO_2_, before starting the experiment and after 8, 24 and 36 h of growth. Experiments were conducted on standard MH medium containing the following per litre: 5 g yeast extract, 10 g soy peptone, 2 g (NH_4_)_2_SO_4_, 0.2 g CaCl_2_∙H_2_O, 0.2 g MgCl_2_∙6H_2_O, 2 g NaCl, 3 g K_2_HPO_4_, 10 g NaCO_3_,1 mg Na_2_S 9H_2_O, supplemented with glucose as C source. The MH medium was supplemented with 10–20–30–40–50 and 60 g/L succinic acid before strain inoculation and cell growth was monitored by diluting the culture with 1 M HCl, to remove NaCO_3_/precipitates, and immediately establish cell concentration as optical density at 600 nm. Every bottle experiment was repeated at least twice.

### HPLC quantification of sugars, organic acids and inhibitors

Broth samples were collected every 2 h during cultivations to follow biomass formation, substrate consumption and product formation. The supernatants obtained after centrifugation were ultrafiltered on 3 kDa centricon devices (Millipore, Bedford, MA, USA) at 12000×*g* and the flow through was analysed for the determination of glucose, xylose and acids produced during growth by HPLC (UHPLC Dionex Ultimate 3000; Thermofisher) on a Alltech IOA-2000 column (500 mm × 6.5 mm ID). Analyses were performed at 40 °C with 0.1% v/v phosphoric acid in water as mobile phase at a flow rate of 0.6 mL/min. Detection was performed via UV absorbance at 200 nm and refraction index (Shodex RI-101 detector, Max auto step 5,1 s, Temperature 32 °C, Rise time 1 s, Polarity plus, Record Range 512 µRIU, Integrator Range 500 µRIU/UV).

### Determination of biomass composition

The materials have been characterized to assess the possibility to obtain the energetic valorization of the residue of the hydrolysis and fermentation process. For this purpose the strain, the *A. donax* hydrolysate used as feedstock, and the residual solid obtained after fermentation, have been characterized by ultimate analysis, i.e. the determination of carbon, hydrogen and nitrogen content (oxygen was calculated by difference) using a CHN LECO CS144 analyser.

For these experiments, about 20 mg of dried and homogenised sample (by grinding) was introduced in the apparatus. The C, H, N content was obtained by burning the sample at 950 °C and measuring the gaseous concentrations using a TCD for N_2_ (previously obtained from catalytic NOx reduction) and two separated NDIR cells for C and H. For the ultimate analysis the protocol ASTM D 5373 has been used. The moisture, the volatile, the fixed carbon and the ash content of the all materials have been determined using a LECO TG701 thermo-gravimetric analyser using about 1.5 g of sample following the ASTM D5142 protocol.

### Performance evaluation of an optimised succinic acid production facility

In this section, by utilizing experimental results obtained from the hydrolysis and fermentation test FB4, which showed the best performance in term of succinic acid yield, a quantified process flow diagram for an optimised biochemical conversion of *A. donax* to succinic acid was defined. The optimised process (OP) provides the use of *A. donax* as the only carbon source, i.e. without the addition of pure glucose as shown during the fermentation test FB4.

The main operation units considered for the proposed OP are that of biomass pre-treatment, hydrolysis, fermentation and purification. The description of the additional units proposed to define the entire succinic acid production process, i.e. biomass pre-treatment and purification units, is as follows:

- The steam explosion process was considered as biomass pre-treatment method as it is a valuable technology to improve the recovery of sugars and other useful compounds from biomass [19];
- The downstream processing (purification unit) involves separation and purification steps that produce a purified succinic acid from the fermentation unit. In particular, it consists of a reactive extraction performed by using tri-n-octylamine in 1-octanol at pH 5, a vacuum distillation and a crystallization process for the highly purified succinic acid production as reported by [20]. Before crystallization, pH was adjusted below 2 with HCl to convert succinate in succinic acid [20].

The experimental results, obtained from the tests of hydrolysis and fermentation (actual process, AP), literature data and those generated by a recently defined environmental assessment tool, the MFA, which is named substance flow analysis (SFA) when it is referred to a specific chemical species, are combined to evaluate the performance of both AP and OP.

The software used to carry out the MFA is the STAN (published by TU Wien, Institute for Water Quality, Resource and Waste Management) that is a freeware that helps to perform material flow analysis according to the Austrian standard ÖNorm S 2096 (Material flow analysis—application in waste management). Each balance is performed with reference to a same block diagram on different layers: one of these is dedicated to the balance of ‘‘goods’’ (in this case, the total mass of reactants and products involved in the AP and OP) and the others to different single substances (i.e. carbon, hydrogen, oxygen, nitrogen, etc.).

The calculation procedure adopted includes the following steps:

- Determination of the amount of the pre-treated *A. donax* utilised in the hydrolysis step of the AP and the transfer coefficients (TC) applying the MFA to the hydrolysis and fermentation units involved in the test FB4. The TCs was calculated as following:
  4 $$TC_{{F,x}} = \frac{{W_{{F,x}} }}{{W_{{F^{{IN}} }} }}$$
  where ${\text{TC}}_{{F_{x} }}$ is the TC of the considered flow, *W*_*F*,*x*_ is the mass of the considered flow and $W_{{F^{\text{IN}} }}$ is the mass of the input flow to the considered process unit.
- Determination of the amount of virgin biomass fed into the pre-treatment unit of the OP on the basis of sugars involved in the AP, i.e. glucose and xylose produced during the hydrolysis step and pure glucose added during the fermentation process. The amount of biomass entering the OP (*W*_*AD*–*OP*_) was calculated using the following equation:
  5 $$W_{{{\text{AD}}-{\text{OP}}}} = \frac{{W_{{{\text{Glu-AP}}}} + W_{{{\text{Glu-Add}}}} + W_{{{\text{Xyl-AP}}}} }}{{X_{{{\text{Glu-AP}}}} + X_{{{\text{Xyl-AP}}}} }},$$
  where *W*_Glu-AP_ and *W*_Xyl-AP_ are glucose and xylose produced during the hydrolysis step of the AP, *W*_Glu-Add_ is the glucose added during the test FB4 and *X*_Glu-AP_ and *X*_Xyl-AP_ are the fractions of glucose and xylose produced during the hydrolysis step of the AP;
- Definition of the quantified flow diagram of the OP that uses biomass as the only carbon source.

In order to ensure similar operating conditions of the two processes (AP and OP), the amounts of the reagents used (MH medium, carbon dioxide, yeast extract and ammonium hydroxide) and the reaction volume were kept constant (same concentration). Also acetic, formic and lactic acid by products were considered in the calculations.

## Results and discussion

### Evaluation of *A. donax* saccharification experiments

Currently, one of the major targets for the biotechnological production of succinic acid is cost reduction through the use of lignocellulosic feedstocks as raw materials. In the present paper a strategy combining steam explosion with enzymatic hydrolysis demonstrated a degree of cellulose and hemicellulose solubilization from *A. donax* biomasses of about 79 ± 8 and 55 ± 5%, according to Eqs. 1 and 2 “Enzymatic hydrolysis of *A. donax*” section, respectively. The concentrations of glucose and xylose found in the hydrolysate, together with the conversion efficiencies, are reported in Table 1. *A. donax* was recently attracting growing interests as renewable material for the production of value-added products, not only for economic aspects, but also for its cultivation ease and status of non-food/waste crop. Giacobbe and collaborators [21] also evaluated the saccharification of *A. donax* pretreated biomass and developed a new purified enzyme cocktail that achieved 62 and 63% conversion of glucan and xylan, respectively. Combined dilute acidic hydrolysis and enzymatic hydrolysis on the recovered *A. donax* solid residue showed a 94 and 70% conversion efficiency of xylans and glucans, respectively [15]. Results obtained in this work are, therefore, in line with those reported in the literature.

**Table 1:** Sugar concentrations and saccharification efficiency after steam pretreatment and enzymatic hydrolysis, calculated according to Eq. 1 and 2

	*A. donax* biomass (Kg dry biomass)	Total volume (L)	Glucose^a^ (g/L)	Xylose^a^ (g/L)	Cellulose conversion (%)	Hemicellulose conversion (%)
FB1	6.4	65.5	33.0	16.6	89.9	58.2
FB2	6.0	61.4	26.5	14.7	72.2	51.5
FB3	6.4	65.5	27.0	14.0	73.6	49.1
FB4	6.4	65.5	29.0	17.0	79.0	59.6
Mean			28.9	15.6	78.7	54.6
SD			2.9	1.4	8.1	5.1

^a^Indicates the concentration of glucose and xylose found in the hydrolysate

### Performance of *B. succiniciproducens* BPP7 on *A. donax* hydrolysates in fed-batch fermentations on the pre-pilot scale

**Fig. 1:**
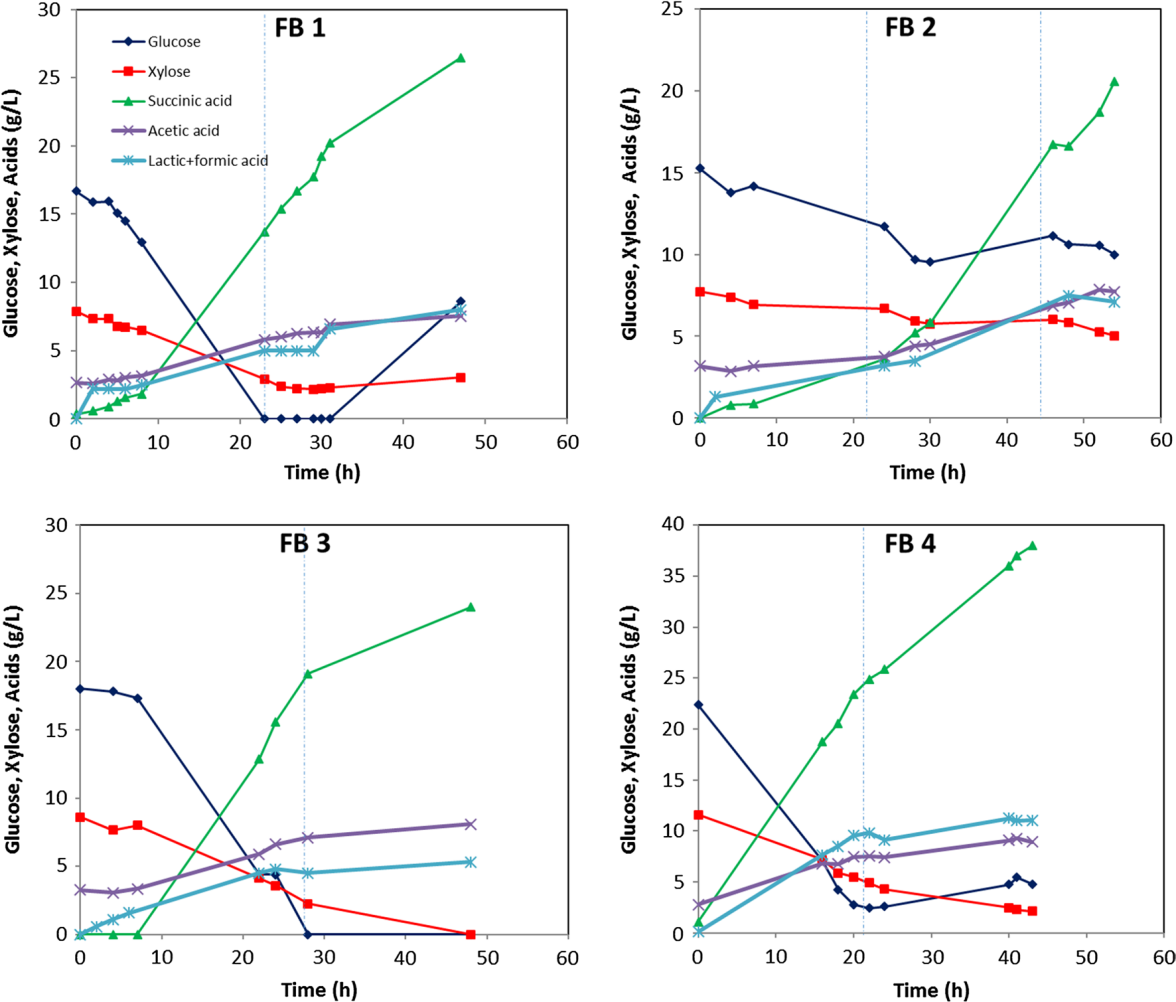
Pilot scale FB experiments. Production of succinic acid from *B. succiniciproducens* in fed-batch experiments on the 150 L scale with an initial working volume of 70 L. The vertical dotted line indicates FB start (In FB2 also the end of the feeding phase is indicated). Feed type: FB1, *A. donax* +glucose + xylose (5:1); FB2, *A. donax* +glucose + xylose (1.9:1); FB3, *A. donax *+ glucose; FB4, pure glucose. Total sugars feed rate: FB1, 1.2 g/L h; FB2, 0.5 g/L h; FB3, 0.2 g/L h; FB4, 0.8 g/L h

Hydrolysed *A. donax* was used for fed-batch experiments on the pre-pilot scale. Different experimental conditions regarding the preparation of the feed solution, the profile used for its administration and the ratio of fed glucose to xylose, were analysed. When using a feed solution composed of *A. donax* supplemented with glucose and xylose (Fed-batch 1 and 2, Fig. 1, Table 2) an average production of 24 ± 4 g/L of succinic acid was achieved in the medium. In both cases, however, a high concentration of glucose and xylose of about 9 ± 1 and 4 ± 1 g/L, respectively, was accumulated in the medium by the end of the experiment (Table 2); the addition during the second experiment (FB2) of a lower amount of total sugars (33 g/L vs 55 g/L), due to the slower feed rate (0.54 g/L h vs 1.20 g/L h), resulted in a 40% higher yield of succinic acid on consumed sugars equal to 0.89 g/g, to the detriment of productivity (0.40 g/L h vs 0.56 g/L h); however, a quite high concentration of glucose and xylose was found throughout the process, still indicating an overload in that conditions (Fig. 1). When further decreasing the feed rate to 0.24 g/L h of *A. donax* supplemented with glucose only (FB 3, Fig. 1, Table 2) no accumulation of glucose and xylose in the medium was observed. Twenty-four g/L of succinic acid were produced after 48 h of growth with a *Y*_SA/Gl+Xy_ of 0.79 g/g and a productivity of 0.5 g/L h (Table 2). The highest concentration of succinic acid, equal to 37 g/L, was reached when adding pure glucose, during the fed-batch phase, at a constant rate of 0.8 g/L h. The process lasted 43 h and resulted in a *Y*_SA/Gl+Xy_ of 0.9 g/g and a productivity of about 0.9 g/L h. However, also under these conditions, the accumulation of 4.78 and 2.11 g/L of glucose and xylose, respectively, was still observed. A similar final concentration of SA (33.8 g/L) was previously obtained in fed-batch experiments on nanofiltered spent sulphite liquor with a slightly lower yield of SA on consumed sugars (0.58 g/g) and productivity (0.48 g/L h) [22]. Overall, *B. succiniciproducens* BPP7 demonstrated a significantly lower sugar consumption rate during the fed-batch phase; in fact, only feeding about 0.24 g/L h of total sugars did not lead to sugar accumulation in the medium. The different ratios of* A.donax*-derived to pure sugars used in FB experiments could not be related to the different fermentation results. It seems, however, that higher glucose:xylose ratios reduced the total acid by-product to succinic acid proportion (Table 3); these data are consistent with those reported by Pateraki and collaborators [22] that observed a higher total acid (TA)/SA ratio, equal to 0.65 g/g, when growing *B. succiniciproducens* on a semidefined medium containing 73% xylose and 11% glucose. The authors also observed increased TA/SA values (0.9–1 g/g) in spent sulphite liquor-based media, indicating that the inhibitors present in lignocellulosic substrates stimulate the production of other acid by-products [22]. Although the use of lignocellulose hydrolysates as sole C source is more suitable for the development of sustainable fermentation processes, concentrated *A. donax* would also contain high concentrations of inhibitory molecules such as acetic acid and phenolic compounds that were previously shown to strongly delay the growth of *B. succiniciproducens* [10]. By comparing FB1 and FB2 it seems that SA production was favoured by a higher glucose:xylose ratio in the feed (5:1, FB1 vs 2:1, FB2 mock *A. donax* ratio) probably due to the faster consumption rate of glucose compared to xylose in co-presence. In FB2, probably the sugar overload and the higher xylose supply also addressed metabolism towards a higher production of lactic and formic acid byproducts as indicated by the ratios reported in Table 3. Overall, the best final titer and productivity of succinic acid were achieved when pure glucose was fed to the culture, indicating that, as for *A. succinogenes,* also *B. succiniciproducens* produces more SA on hydrolysates that contain more glucose than xylose [23, 24].

**Table 2:** Comparison of fed-batch processes performed by growing *B. succiniciproducens* BPP7 on the 150 L scale

	SA (g/L)	Cells (*g*_cdw_/L)	Time (h)	Tot sugars *A. donax* (g/L)	Tot pure sugars (g/L)	Ratio *A. donax*/pure sugars	Ratio Gl/Xy	Tot added sugars (g/L)	*Y*_SA/gl + xy_ fed (g/g)	*Y*_SA/gl + xy_ cons (g/g)	*r*_proc_ (g/L h)	Gl_acc_ (g/L)	Xy_acc_ (g/L)	Feed type	Sugar feed rate (g/L h)
FB1	26.5	4.0	47	26.3	28.9	0.9	3.3	55.2	0.5	0.6	0.6	8.6	3.0	*A. donax *+ Gl +Xy	1.2
FB2	21.0	3.9	54	23.9	9.8	2.4	1.9	33.7	0.6	0.9	0.4	10.0	5.0	*A. donax *+ Gl +Xy	0.5
FB3	24.0	2.8	48	27.1	3.2	8.4	2.5	30.3	0.8	0.8	0.5	0.0	0.0	*A. donax *+ Gl	0.2
FB4	37.0	4.0	43	23.0	19.9	1.2	4.0	42.8	0.8	0.9	0.9	4.8	2.1	Gl	0.8

*Y*_SA/gl+xy_ fed, is the yield of succinic acid on fed glucose and xylose; *Y*_SA/gl+xy_ cons, is the yield of succinic acid on consumed glucose and xylose; *r*_proc_, is the volumetric productivity on the entire process; Gl_acc_ and Xy_acc_, are the concentrations of glucose and xylose found in the broth at the end of the process

**Table 3:** Overall process efficiency calculated according to Eq. 3

	SA (g/L)	*V*_s_ (L)	*V*_h_ (L)	*A. donax* glucose added (g/L)	*A. donax* xylose added (g/L)	Pure glucose added (g/L)	Pure xylose added (g/L)	LA + FA/SA (g/g)	AA/SA (g/g)	SA/TA (g/g)	TA/SA (g/g)	Fermentation efficiency (%)
FB1	26.5	84.3	43.0	17.9	8.5	24.4	4.4	0.30	0.18	2.06	0.48	46.9
FB2	21.0	72.0	36.5	15.8	8.1	6.3	3.6	0.36	0.22	1.77	0.56	67.0
FB3	24.0	69.3	42.9	18.3	8.8	3.2	–	0.22	0.20	2.37	0.42	49.2
FB4	37.0	82.0	35.0	14.5	8.5	19.9	–	0.29	0.17	2.16	0.46	75.4

*V*s, indicates the volume of supernatant recovered at the end of the fermentation process; *V*_h_, is the volume of hydrolysate added to the medium; SA, succinic acid; LA, lactic acid; FA, formic acid; AA, acetic acid; TA, LA + FA + AA

A global mass balance was performed on all fermentation experiments considering the fractions of cellulose and hemicelluloses present in the treated dry biomass and the maximal theoretical yield of succinic acid on glucose and xylose consumed (Eq. 3), resulting in a fermentation efficiency ranging between 47 and 75% (Table 3). The fermentability of *A. donax* was previously evaluated for the production of second-generation bioethanol demonstrating an enzymatic conversion efficiency of the cellulose (contained in already partially delignified cellulignin) to glucose of about 52%, and a fermentation efficiency from glucose to ethanol of about 40% [25]. Compared to the previously developed batch process in our lab [10], the concentration of succinic acid, the volumetric productivity (*r*) and the *Y*_SA/Gl+Xy_ of the process were improved by 2.4-, 2.7- and 1.3-fold, respectively. In batch conditions on corn stover *B. succiniciproducens* produced 30 g/L of product with a yield of about 0.69 g/g on consumed sugars [11]. Recently, an efficient fed-batch process with immobilized cell cultures reached 45 g/L of succinic acid in 80 h from spent sulphite liquor [12]. Productivity, however, was about 55% lower compared to that described in the present work.

### Succinic acid growth inhibition

**Fig. 2:**
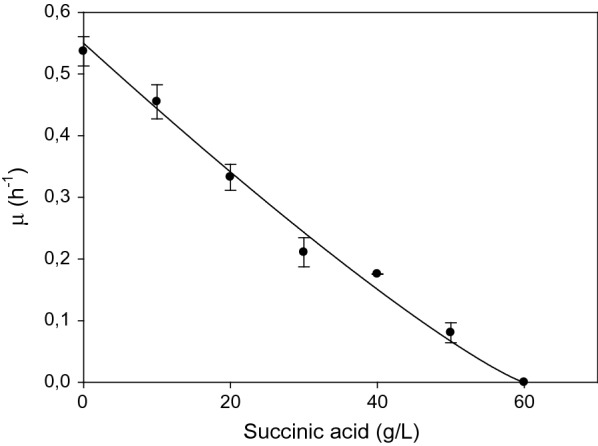
Product inhibition. Evaluation of succinic acid inhibition on the growth of *B. succiniciproducens* in bottle experiments with an initial glucose concentration of 10 g/L, pH 6.5 and temperature of 37 °C

Bottle experiments on MH medium supplemented with increasing concentrations of succinic acid in the medium showed a decrease of the total biomass produced and of the specific growth rate (*μ*), calculated as linear regression of OD measurements over time during the first 6 h of growth. The experimental data were fitted to the Luong-model [26, 27] exponential inhibition equation:

6 $$\mu = \mu_{max} \left( {1 - \frac{P}{{P_{max}}}} \right)^{{n_{p} }} ,$$

where *μ*_max_ is the maximum specific growth rate calculated in the absence of succinic acid in the medium, *P* is the concentration of product (succinic acid), *P*_max_ is the critical product concentration (when *P *=* P*_max_*, μ *= 0) and n_p_ is the inhibition constant (for non competitive inhibition *n*_*p*_ > 0). We found a *P*_max_ of 60 g/L and a *μ*_max_ equal to 0.55 h^−1^ determined experimentally (Fig. 2). A regression coefficient (*R*^2^) equal to 0.98 was found analysing data with Sigma plot.

Lin and collaborators proposed a kinetic model for *A. succinogenes* that considers substrate and product inhibition and found a threshold for SA inhibition equal to 104 g/L [28]. This characterization of the strain physiology was not performed for *B. succiniciproducens* up to date, and it is interesting to notice that a 60% reduction of the growth rate was already observed in the presence of 30 g/L of succinic acid in the medium.

### Performance assessment of an optimized process for SA production

This section evaluated another aspect of industrial development that was not considered up to date; in fact, only few works are addressed to the performance assessment of the whole SAPP.

**Fig. 3:**
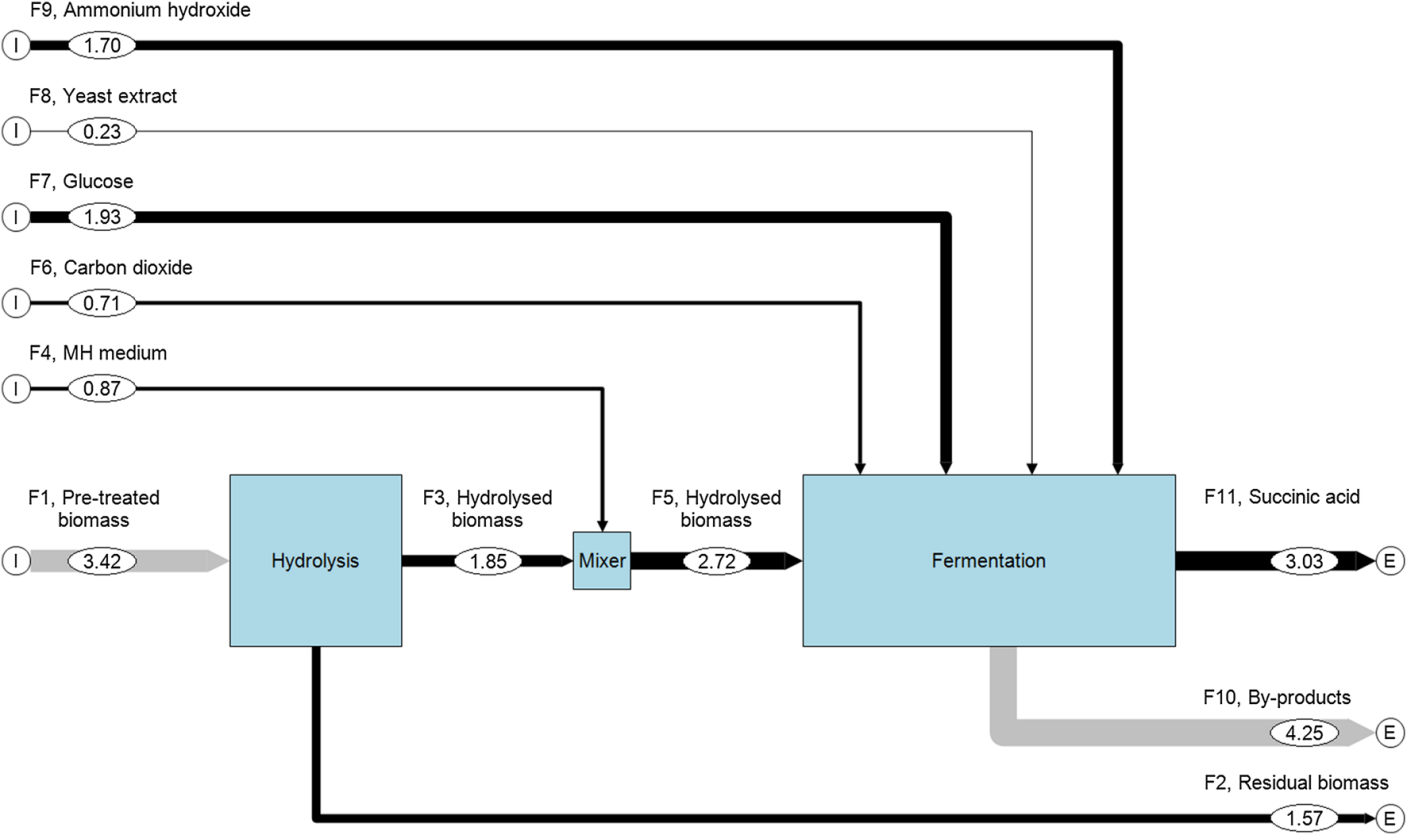
Quantified flow diagram as a result of the MFA applied to the hydrolysis and fermentation units involved in the FB4 test

In particular, the experimental results obtained from the FB4 experiment, literature data and those generated by the MFA and by the characterization of specific process streams were combined to evaluate the performance of a facility that encloses the main steps involved in the SA production process, i.e. biomass pre-treatment, hydrolysis, fermentation and purification. The OP proposed uses *A. donax* as the only carbon source. The MFA analysis consists in a systematic assessment of the flows and stocks of materials and elements within a system defined in space and time. In particular, it connects the sources, the pathways and the intermediate and final sinks of each species in a specific process [29]. These characteristics make MFA a flexible decision support tool attractive for different application fields, as showed by its utilization in process evaluation of waste thermochemical conversion and recycling options [30, 31] and in waste management planning [32]. Figure 3 shows the layer of total mass as a result of the MFA applied to the FB4 experiment, i.e. the quantified block diagram on dry basis of the hydrolysis and fermentation units. The inputs to the process units are: pre-treated biomass, MH medium, carbon dioxide, glucose, yeast extract and ammonium hydroxide. The output streams are: non-hydrolysed biomass, succinic acid and by-products (unconverted sugars, other acids, microorganism, inorganics, etc.) produced during the fermentation step. Each flow in input to or in output from a specific unit is identified with a black arrow if the specific data were measured, or by a grey arrow if the data were obtained by means of the material balance of the MFA. The quantified block diagram displayed in Fig. 3 shows that the TC related to the flow *F*_3_ (TC_*F*,3_) is 0.54. Since the overall pre-treated biomass was used for the hydrolysis process, the combined efficiency of the pre-treatment and hydrolysis processes is 54% (1.85 kg of hydrolysed biomass). Thus, TC_*F*,2_ is 0.46; this means that the unconverted biomass is 1.57 kg. As a consequence, the amount of the pre-treated biomass, which corresponds to the virgin biomass fed to the process, is 3.42 kg. The output from the fermentation unit is 7.28 kg. During this process 3.03 kg of SA and 4.25 kg of by-products are generated.

**Fig. 4:**
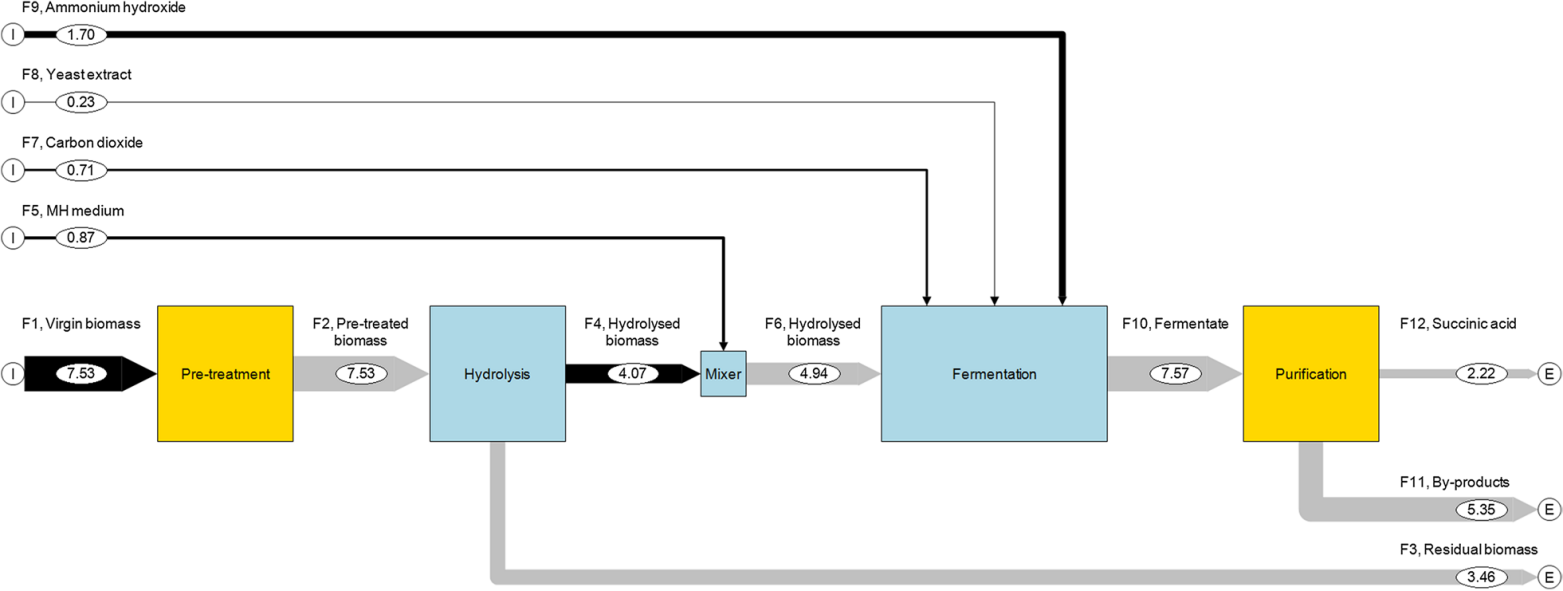
Quantified flow diagram as a result of the MFA applied to the OP

As previously mentioned, the feedstock cost of bio-chemicals production is one of the main ones of the entire manufacturing process; therefore making SA production more economically and environmentally sustainable requires the use of raw materials, such as lignocellulosic biomass, as only carbon source. For this reason, we also evaluated a process based on the use of *A. donax* as only carbon source by translating the amount of pure glucose added during the developed FB fermentation process into additional virgin biomass (Fig. 4). The application of the Eq. 4 shows that 7.53 kg of virgin biomass (i.e. the flow *F*_1_ of Fig. 4) should be pre-treated and hydrolysed to produce the total amount of sugars (3.54 kg) necessary in the AP (sugars generated during the hydrolysis step: 1.02 kg of glucose and 0.59 kg of xylose; glucose added during the fermentation process: 1.93 kg). The flows *F*_3_ and *F*_4_ are generated by utilising the TCs of the hydrolysis unit of the AP (0.46 and 0.54 for *F*_3_ and *F*_4_, respectively), while the inputs F_5,7–9_ are the same of Fig. 3.The last process unit considered in the OP involves purification and separation steps which generate a highly purified SA stream, according to literature data [20]. In particular, it consists of a reactive extraction, a vacuum distillation and a crystallization process that ensure a SA purity of 98% and a yield of 73.1%, as indicated by the authors. As a result, the flow *F*_12_ is equal to 2.22 kg (constituted of 98% of succinic acid), while the flow *F*_11_ consist of 5.35 kg of process by-products (unconverted sugars, other acids, microorganism, inorganics, etc.) and of the unrecovered SA. The main results of the MFA application to the OP proposed are that this process yields 29.48% of purified SA per kilogramme of virgin biomass. On the other hand, the by-products amount to 79.80% of the total input. The 48.46% of this should be disposed (*F*_11_), while the 31.34% is unconverted biomass of the hydrolysis step (*F*_3_). Considering the proposed OP in a commercial scale, this process residue (composed mainly of lignin) could represent a valuable feedstock for bioenergy and biochemicals’ production in a view of biorefinery complexes development [33]. It must be highlighted that the considered OP utilized the highest conversion factor of sugars to SA obtained from the fermentation test FB4. As can be deduced from Table 3, the mass conversion factors range from about 50 to 80%. Considering, as a conservative evaluation of SA production, the conversion factor obtained using the data from all the experiments that we conducted (FB1-2-3-4), the yield of SA decreases from 30 to 25% (Additional file 1: Figure S1), which demonstrates the robustness of our chosen MFA approach and confirms the validity of using *B. succiniciproducens* with *A. donax* as substrate with the objective of achieving a sustainable process for the production of succinic acid.

### Evaluation on the valorisation of residual and global carbon balance

The results of proximate and ultimate analysis are reported in Table 4. Both the hydrolysed *A. donax* and the solid after fermentation show a very high moisture content, while as expected the presence of moisture of the microorganism is reduced according to the nature of the material. The concentration of the N in the solid coming from the fermentation step is about two times higher than that of the starting material while a reduction of the ash concentration is observed. The H/C and O/C ratio evaluated for the hydrolysed *A. donax* (Table 4) and compared with those reported by Chen and collaborators [34] (1.67, 1.60 and 1.19–1.53 and to 0.83, 0.8 and 0.47–0.36 for cellulose, hemicellulose and lignin, respectively) show that in the sample a fraction of cellulose ad hemicellulose is still present in addition to the lignin.

**Table 4:** Proximate and ultimate analysis

Proximate analysis, %_wt, ar_	Hydrolysed *A. donax*	*B. succiniciproducens*	Solid after fermentation
Moisture	70.88	5.5	76.43
Volatile matter	19.37	79.3	19.44
Fixed carbon	6.80	7.6	2.81
Ash	2.95	7.6	1.32
Ultimate analysis, %_wt, ar_			
C	12.78	39.15	11.40
H	0.60	6.03	1.63
N	0.88	10.80	2.78
O (by diff.)	11.91	30.92	6.44
H/C (molar ratio)	0.56	1.85	1.71
O/C (molar ratio)	0.70	0.59	0.42
C/ash (weight ratio)	4.33	5.15	8.64

**Fig. 5:**
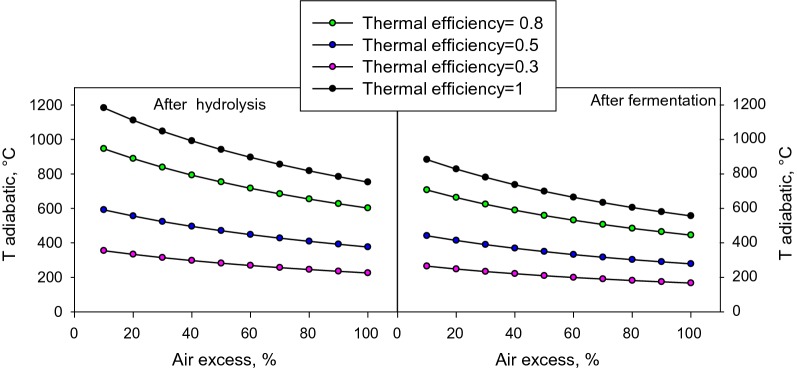
Adiabatic temperature as function of the excess air factors and for different thermal efficiencies

On the basis of the proximate and ultimate analysis a preliminary analysis on the reliability of the combustion process using both the hydrolysed *A. donax* and the residual of the fermentation as fuel was carried out. More specifically, the adiabatic temperature, i.e. neglecting the external heat dispersion, considering different excess air factors and assuming different conversion efficiency (Fig. 5) has been evaluated. Curves in Fig. 5 indicate that the adiabatic temperature is almost always lower than 800 °C suggesting that an autothermal combustion process is not sustainable. In particular, it is possible to obtain an adiabatic temperature higher than 800 °C only operating the reactor at very low air excess factors and considering a thermal efficiency not lower than 0.8 using the starting material (the hydrolysed *A. donax*). If the residue of the fermentation step would be used as fuel, a more restrictive condition should be adopted to obtain an adiabatic temperature of 800 °C, more specifically an excess factor lower than 10% and only for the thermal efficiency equal to one can be used. The latter result is in agreement with the further conversion of biomass in the fermentation step with a consequent reduction of the energy content of the residual material. The results suggest that a pre-treatment to reduce the moisture content is necessary before the combustion process. In the utilization of the residual materials as fuel for a combustion process the other critical point is the high concentration of N in the samples since a high concentration of nitrogen oxides (fuel NOx) is expected. A basic calculation of the amount of NO_2_ equivalent emitted during the combustion was done. In particular, the concentration of NO_2_ was estimated considering a fuel nitrogen conversion between 10 and 40%, in agreement with previous results [35]. The authors performed fluidized bed combustion experiments with a high N concentration fuel such as olive husk and considered the air excess necessary to obtain a concentration of oxygen equal to 11 vol% at the exit of the gas. The value of NO_2_ concentration in the emitted gas obtained considering a conversion of only 10% (1378 and 3528 mg/Nm^3^ for hydrolysed *A. donax* and the residual of fermentation, respectively) is exceeding the threshold prescribed by the Italian normative (500 mg/Nm^3^ of NO_2_ at an O_2_ concentration of 11% vol. D.Lgs.33 Aprile 2006 n. 152) suggesting that a post treatment section for the abatement of NOx should be included.

## Conclusion

Succinic acid production from *B. succiniciproducens* on *A. donax* was studied with different FB experiments on the pre-pilot scale, with a highest process efficiency of about 75% considering the hydrolysis and fermentation process units. Best experimental results were analysed by material flow analysis to evaluate another aspect of industrial development and assess the performance of the entire production process. Results indicated the obtainment of 88.5% succinic acid per kg of biomass used and 52% of product on the total output generated. Considering an optimized process that uses *A. donax* as only C source, data indicated a potential yield of about 30% of product and an unconverted residue of 31% mainly composed of lignin, a potentially valuable feedstock for bioenergy and biochemicals’ production.

## Additional file

**Additional file 1: Figure S1.** Quantified flow diagram as a result of the MFA applied to the OP by using an average fermentation efficiency of all fermentation tests performed (FB1-2-3-4).

## Abbreviations

SA: succinic acid
FB: fed-batch
MFA: material flow analysis
OP: optimized process
SAPP: succinic acid production process
AP: actual process
