# Supplementary figures and images for: Improved production of succinic acid from Basfia succiniciproducens growing on A. donax and process evaluation through material flow analysis
Source: Biotechnol Biofuels. 2019 Feb 4;12:22. doi: 10.1186/s13068-019-1362-6 (PMC6360672; doi:10.1186/s13068-019-1362-6)

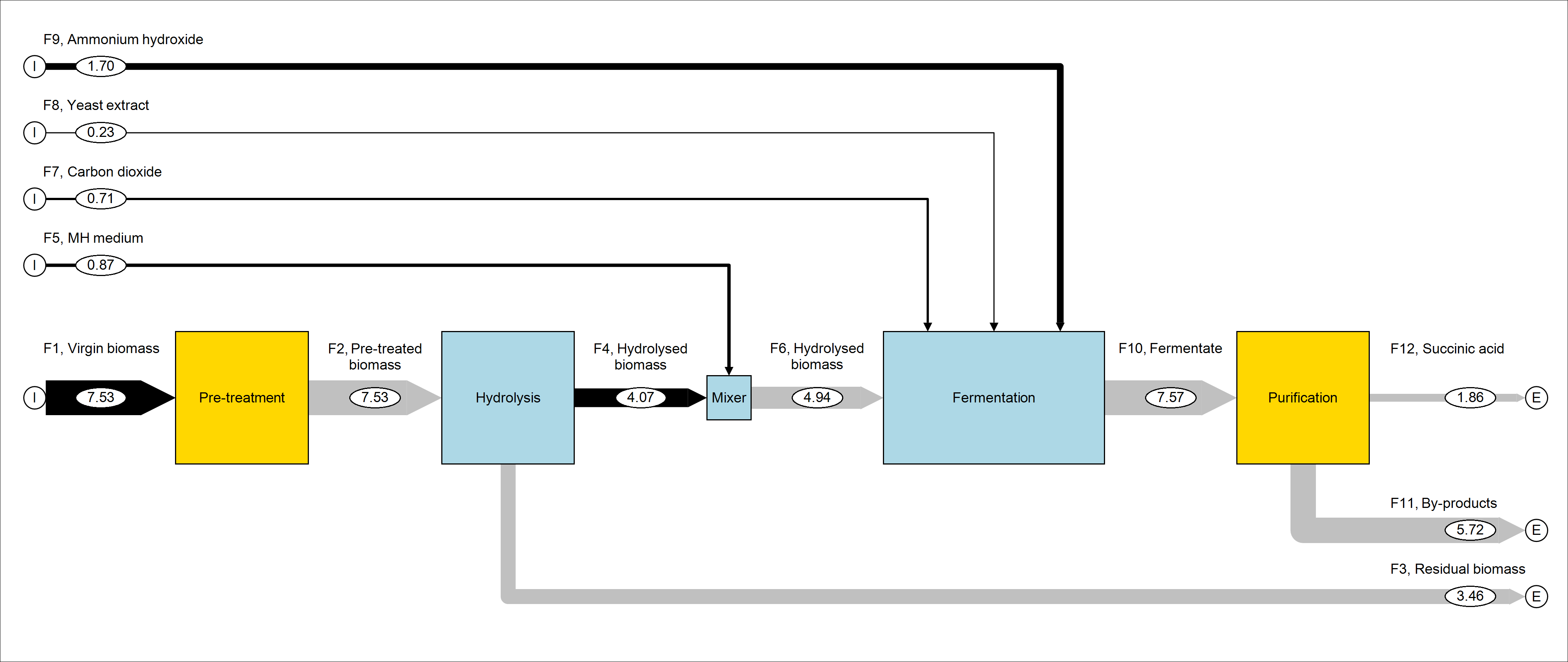

Supplement: Supplementary file 1 — Additional file 1: Figure S1. Quantified flow diagram as a result of the MFA applied to the OP by using an average fermentation efficiency of all fermentation tests performed (FB1-2-3-4). [file 13068_2019_1362_MOESM1_ESM.docx]
